# MicroRNA-92a as a Potential Biomarker in Diagnosis of Colorectal Cancer: A Systematic Review and Meta-Analysis

**DOI:** 10.1371/journal.pone.0088745

**Published:** 2014-02-14

**Authors:** Xin Yang, Zongyue Zeng, Yixuan Hou, Taixian Yuan, Chao Gao, Wei Jia, Xiaoyan Yi, Manran Liu

**Affiliations:** 1 Key Laboratory of Laboratory Medical Diagnostics designated by Chinese Ministry of Education, Chongqing Medical University, Chongqing, China; 2 Experimental Teaching Center of Basic Medicine Science, Chongqing Medical University, Chongqing, China; Sapporo Medical University, Japan

## Abstract

**Introduction:**

Previous studies demonstrated that MicroRNA-92a (miR-92a) was significantly differential expressed between colorectal cancer (CRC) patients and control cohorts, which provide timely relevant evidence for miR-92a as a novel promising biomarker in the colorectal cancer patients. This meta-analysis aimed to evaluate potential diagnostic value of plasma miR-92a.

**Methods:**

Relevant literatures were collected in PubMed, Embase, Chinese Biomedical Literature Database (CBM), Chinese National Knowledge Infrastructure (CNKI) and Technology of Chongqing (VIP), and Wan Fang Data. Sensitivity, specificity and diagnostic odds ratio (DOR) for miR-92a in the diagnosis of CRC were pooled using random effects models. Summary receiver operating characteristic (SROC) curve analysis and the area under the curve (AUC) were used to estimate the overall test performance.

**Results:**

This Meta-analysis included six studies with a total of 521 CRC patients and 379 healthy controls. For miR-92a, the pooled sensitivity, specificity and DOR to predict CRC patients were 76% (95% confidence interval [CI]: 72%–79%), 64% (95% confidence interval [CI]: 59%–69%) and 8.05 (95% CI: 3.50–18.56), respectively. In addition, the AUC of miR-92a in diagnosis CRC is 0.7720.

**Conclusions:**

MicroRNA-92a might be a novel potential biomarker in the diagnosis of colorectal cancer, and more studies are needed to highlight the theoretical strengths.

## Introduction

Colorectal cancer (CRC) is one of the most common malignancies. With an estimated 1.2 million new cases and over 6 hundred thousand deaths each year, colorectal cancer (CRC) is the fourth most common cancer leading causes of cancer-related mortality worldwide, the third most familiar diagnosed cancer in males and the second in females [1]. Even though the disease develops slowly from premalignant to invasive carcinoma, the prognosis is extremely unsatisfactory due to its diagnosis at an advanced stage [2]. Fortunately, there is evidence that screening of early-stage CRC allows surgical removal of cancer precursor lesions and potentially reduces mortality of the disease [3]. To detect early-stage cancer, several CRC screening strategies, including fecal occult-blood testing (FOBT) and colonoscopy, have been implemented for years. However, the fecal occult-blood testing (FOBT), which is currently the most available noninvasive screening tool, has the limitation of low sensitivity and requires punctilious dietary restriction [4]. On the other hand, as the gold standard method for early detection of CRC, colonoscopy has been rejected because of its invasive nature and the high cost [5]. As a result, a new non-invasive approach is urgently needed to improve the detection of early-stage CRC.

Fortunately, the discovery of microRNAs opened a new window for the early diagnosis of cancer by a non-invasive detection. MicroRNAs (miRNAs) are a class of evolutionarily conserved and small non-coding RNA molecules that regulate a variety of critical cellular processes, including development, differentiation, proliferation, apoptosis and metabolism [6]. Although the natural mechanisms of the dysregulation of miRNAs are far from being completely understood, miRNAs have been demonstrated to play important roles in tumor formation and progression, acting themselves as oncogenes or tumor suppressors and affecting diagnosis, staging, progression, prognosis and treatment for human cancers [7], [8].

During the past decades years, studies have shown that miRNAs expression are significantly various between tumor tissue and normal tissue [8], and these tumor-associated miRNAs have been detected in the blood from cancer patients [9], [10]. The previous studies have revealed different kinds of cancer have distinct miRNA profiles [11]–[14]. In 2009, Ng *et al.* [15] first reported that miR-92a, belonging of miR-17–92 cluster, was significantly upregulated in plasma of colorectal cancer patients compared to healthy individuals, suggesting that miR-92a could be a potential non-invasive molecular for CRC screening. Following, increasing researchers devote themselves to the clinical value of miR-92a in CRC [16]–[20].

To understand whether the miR-92a could serve as a diagnosis biomarker for CRC, we did the systematic review and meta-analysis by using pool of published literatures searched from several authoritative electronic databases without constraints of publication date, and the inception of data sources was published at February 14, 2006. Our data showed that microRNA-92a may be a novel potential biomarker in the diagnosis of colorectal cancer.

## Materials and Methods

### Data Sources and Search Strategy

All relevant articles, limited in title and abstract, were searched via following electronic databases: PubMed, Embase, Chinese Biomedical Literature Database (CBM), Chinese National Knowledge Infrastructure (CNKI) and Technology of Chongqing (VIP), and Wan Fang Data up to November 29, 2013. No restriction was used on language, year of publication and publishing status. The keywords employed for literature retrieval included: (1) Colorectal or colon or colonic or rectal or rectum; (2) Cancer or tumor or tumour or carcinoma or neoplasm or carcinomata; (3) miR-92 or microRNA-92 or hsa-mir-92 or miR-92a or microRNA-92a or hsa-mir-92a. In addition, we also manually searched the references from included articles and relevant published reports.

### Inclusion and Exclusion Criteria

All the studies were carefully decided by two investigators (X.Y and Z.Y.Z) independently based on titles and abstracts, and then found full text for any potential eligibility. Any disagreement was resolved by fully discussion to consensus. Furthermore, if necessary, we turned to the original authors for missing data. Each inclusion article must meet to following criteria: (1) The diagnosis of CRC was based on colonoscopy or histological examination; (2) The matched control individuals were included with a recent negative result of colonoscopy and without a personal history of any types of cancer; (3) All blood samples were collected prior to colonoscopy and without any treatment; (4) The researchers assessed the miR-92a in blood sample alone; (5) The studies should contain the data of sensitivity, specificity (or the possibility of deriving such values from the data), and a clear cut-off value; (6) Only the study with more than 20 cases and matched controls were included. All the studies were excluded if they had any of the following items: (1) Duplicate publications; (2) Letters, editorials, meeting abstracts, case reports and reviews; (3) Unqualified patients and control subjects, as well as their blood samples; (4) Insufficient data. If the same author reported their results acquired from the overlapping population or multiple published data in the different works, only the nearest or the most complete report was included.

### Data Extraction

Two investigators (X.Y and Z.Y.Z) perused the full texts of included studies and extracted the following data independently: authors, country, journal, year of publication, study design, number and characteristics of patients and controls respectively, assay method of the markers, cutoff values and raw data for fourfold table and so on. Disagreements were solved by fully discussing with the third senior investigator to reach a consensus.

### Quality Assessment

The quality of each study was assessed independently by two investigators (X.Y and Z.Y.Z) according to the QUADAS-2 (Quality Assessment of Diagnostic Accuracy Studies 2). The QUADAS-2 is recognized as an improved, redesigned tool which comprises 4 key domains (patient selection, index test, reference standard, and flow and timing) supported by signaling questions to aid judgment on risk of bias, rating risk of bias and concerns about applicability as “high”, “unclear” and “low,” and handling studies in which the reference standard consists of follow-up [21].

### Statistical Analysis

All statistical analyses were performed by Meta-DiSc and STATA 12.0 statistical software [22]. All accuracy data from each study (true positives, false positives, true negatives and false negatives) were extracted to obtain pooled sensitivity, specificity, positive likelihood ratio (PLR), negative likelihood ratio (NLR), positive predicted value, negative predicted value, diagnostic odds ratio (DOR) and their 95% confidence interval [95% CI], simultaneously, generate the summary receiver operator characteristic (SROC) curve and calculate the area under the curve (AUC). The sensitivity, specificity, positive and negative predicted value, diagnostic odds ratio of miR-92a were presented as forest plots. Moreover, the heterogeneity between the studies caused by threshold effect was quantified using Spearman correlation analysis. The Non-threshold effect was assessed by the Cochran-Q method and the test of inconsistency index (I^2^), and a low p-value (≤0.05) and high I^2^ value (≥50%) suggest presence of heterogeneity by caused Non-threshold effect. If the Non-threshold effect existed, meta-regression would be used to find out the sources. For publication bias, all eligible studies were assessed by Begg’s test and Egger’s test using STATA 12.0 statistical software. The *P* value with less than 0.05 shows a result of statistical significance.

## Results

### Data Selection

**Figure 1:**
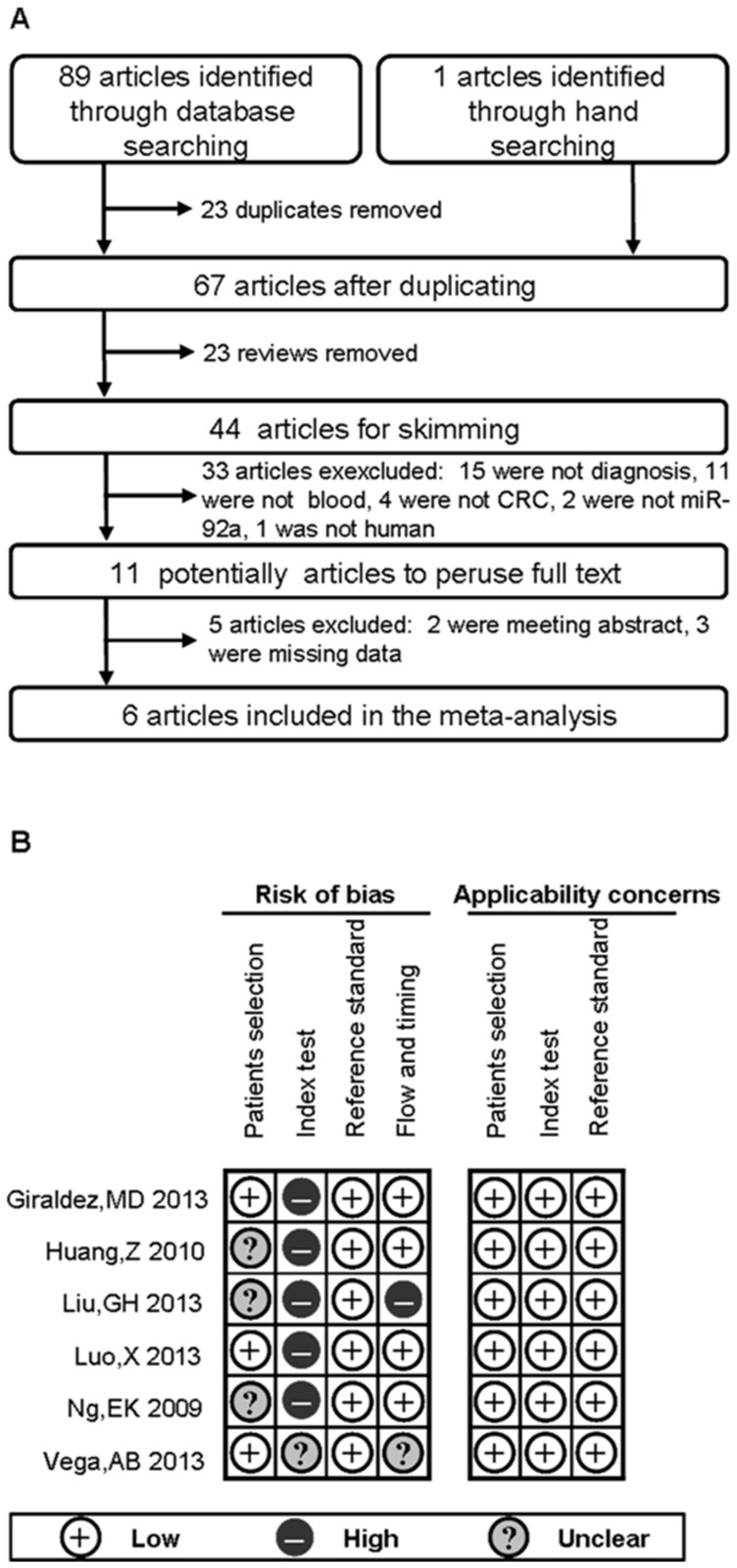
The Flow diagram and quality assessment based on the eligible studies. (A) Flow diagram of study selection process. (B) Quality assessment of the included studies by QUADAS-2. It summarized “risk of bias” and “applicability concerns” through judging each domain for each included study. It shows the major biases concentrated upon the “patient selection” and “index text”.

Ninety literatures were primitively identified according to the literature search strategy from databases and hand searching (Figure 1A). Following the titles and abstract search of these, 23 duplicates and 23 reviews were removed. Of these remained 44 literatures, their full-text versions were retrieved. Of these, 15 studies were not diagnosis, 11 were not used blood sample, 4 were not CRC, 2 were not evaluated miR-92a, and 1 was not on human cancer, so all 33 of these studies were excluded from further analysis. 11 literatures were considered to be potentially eligible were retrieved for full text perusal. Among the 11 cohort studies, an additional 5 studies were excluded from further analysis due to the 2 meeting abstracts [23], [24] and 3 data absence [25]–[27]. Thus, six high-quality cohort studies from independent research group met the eligibility criteria including the informed consent from participants for this systematic review and meta-analysis [15]–[20]. Besides, we sent 4 of e-mails to the authors to request for more details about sensitivity, specificity and AUC, only 1 author give us his response [20], 1 author claimed that the combined index, not the single index of miRNAs has been detected in their study [25], and 2 author had no response to us [26], [27].

### Study Characteristics

All of these eligible literatures were published from 2009 to 2013, accumulating 521 CRC patients and 379 healthy controls. Colonoscopy was considered as gold standard to diagnose the CRC. The study characteristics, including the first author, publish year, country, the numbers of patients and controls, mean age, TNM stage, assay type, internal control, cut-off value, sensitivity, specificity and AUC, are listed in Table 1.

**Table 1:** Main characteristics of studies include in this systematic review and meta-analysis.

First author (year)	Country	Patients/controls	Mean or median Age (yr)	TNM (I/II/III/IV)	Assay type	Internal control	Cut-off values	SE^a^ (%)	SP^b^ (%)	AUC^c^
Ng, EK(2009)	China	90/50	71	6/34/23/27	qRT-PCR,2^−ΔΔct^	RNU6B	240	89.00	70.00	0.8850
Huang, Z(2010)	China	100/59	61	27/25/38/10	qRT-PCR,2^−ΔΔct^	MiR-16	1.231	84.00	71.20	0.8380
Giraldez, MD(2013)	Spain	21/20	72.5	4/8/6/3	qRT-PCR.−Δct	MiR-16	0.2972	95.00	65.00	0.8571
Luo, X(2013)	Germany	80/144	68	22/25/26/5/2*	qRT-PCR.−Δct	MiR-16	2.87	68.21	46.40	0.5609
Liu, GH(2013)	China	200/80	50.09	18/96/64/22	qRT-PCR,2^−ΔΔct^	MiR-16	0.00017	65.50	82.50	0.8470
Vega, AB(2013)	Spain	30/26	64.1	0/0/30/0	qRT-PCR,2^−ΔΔct^	MiR-16 MiR-103 MiR-let-7a	NR^#^	67.00	58.00	0.6350

*2 individuals were not specified by TNM stage.

#: not report. a: sensitivity. b: specificity. c: The area under the curve.

### Quality Assessment

The quality of the included studies was assessed using QUADAS-2 quality assessment. As shown in Figure 1B, all of the 6 inclusions are belong to upper middle quality. However, a major bias was found in these included studies. In general, the major biases of these eligible studies were concentrated upon the “patient selection” and “index text”.

### Heterogeneity and Threshold Effect

**Figure 2:**
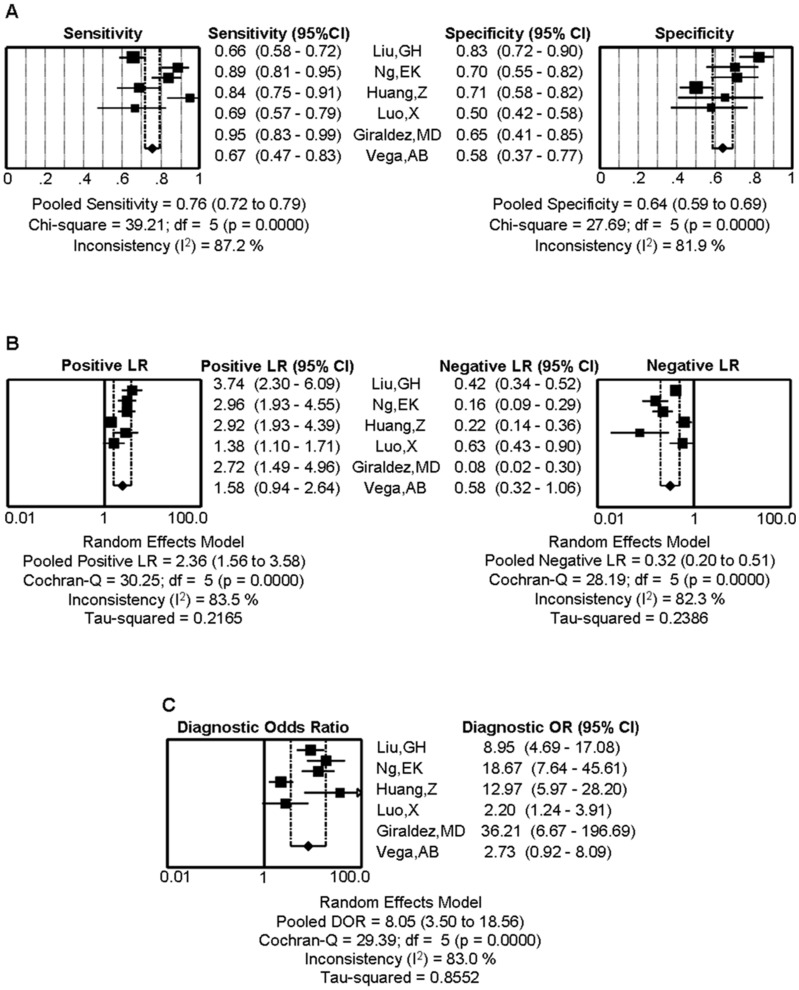
The forest plots show the pooled diagnosis index of miR-92a for the diagnosis of CRC. The point efficiencies from each study are shown as squares and the pooled efficiencies are shown as diamond. Degree of freedom is abbreviated as df. Inconsistency is used to quantify the heterogeneity caused by non-threshold effect. Of these studies, random effects model was used to pool these data. (A) Sensitivity and specificity, (B) PLR and NLR, (C) DOR, and their 95% CI are displayed respectively, which suggests miR-92a might be a potential noninvasive diagnosis biomarker of CRC.

The heterogeneity between the studies is a critical key to understand the possible factors that influence accuracy estimates, and to evaluate the appropriateness of statistical pooling of accuracy estimates from various studies [22]. In order to assess whether the heterogeneity of miR-92a are amongst the eligible studies, we first calculated the correlation coefficient and *P* value between the logit of sensitivity and logit of 1-specificity by using Spearman test to exclude the threshold effect. As a result, the Spearman correlation coefficient was 0.143 and the *P* value was 0.787 (>0.05), indicating that there was no heterogeneity from threshold effect. Due to the non-threshold effect being another key to the heterogeneity between the studies, the inconsistency index (I^2^) was employed. The I^2^ in the forest plot of diagnosis index was more than 50% (as shown in Figure 2) that suggested the heterogeneity caused by non-threshold effect was existed among these studies.

### Data Analysis

**Figure 3:**
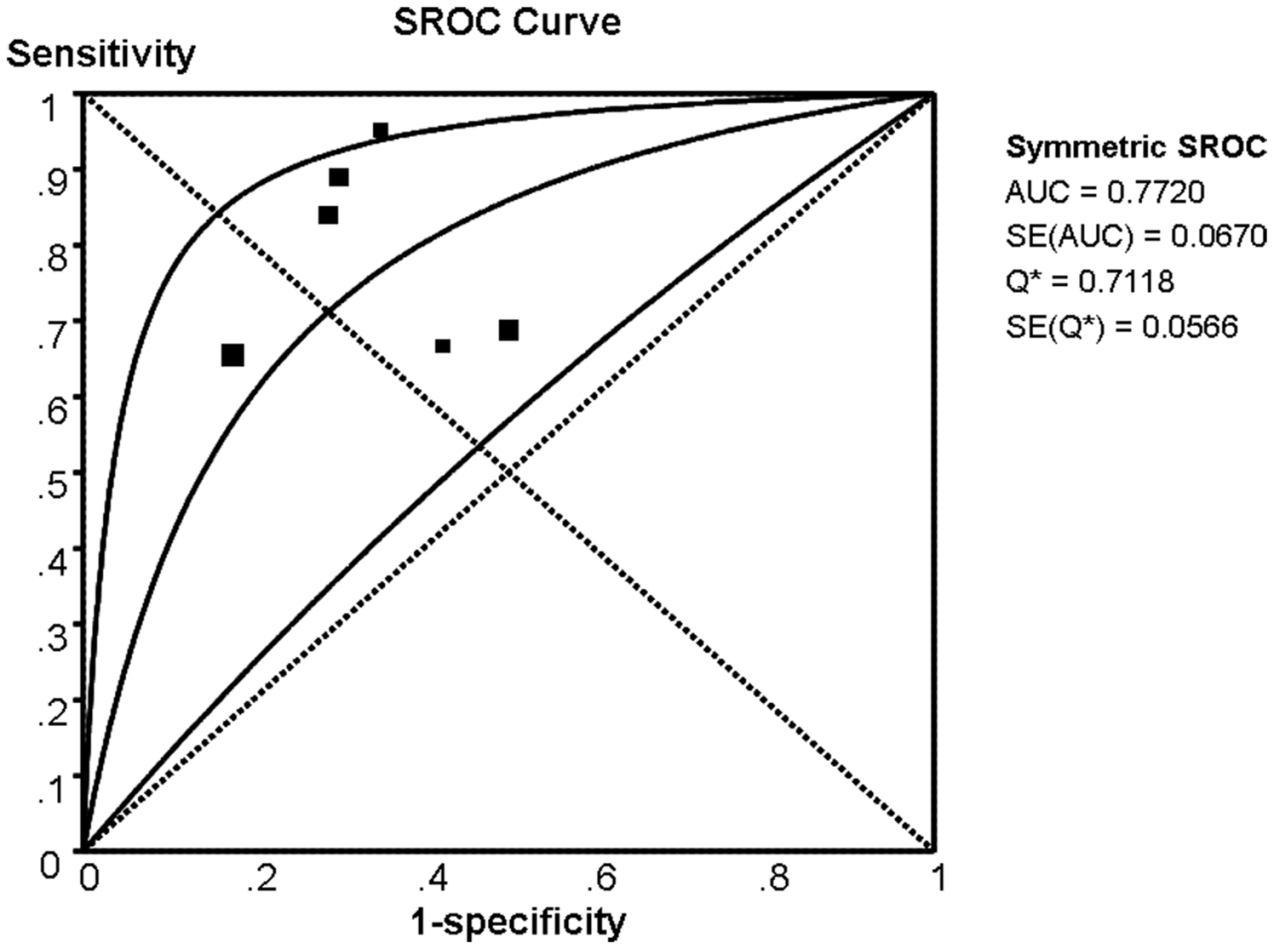
Summary receiver operating characteristic curves (SROC) of miR-92a describes the diagnostic performance. Every square stands for a study. The SROC curve is symmetric and the AUC is 0.7720, which intimates a moderate diagnostic accuracy for diagnosing CRC.

Because of the potential heterogeneity caused by non-threshold effect was among these studies, the random effect model was used to estimate overall performance of miR-92a in diagnosis CRC. For miR-92a, the sensitivity, specificity, PLR, NLR and DOR of 6 included studies were performed by forest plots (Figure 2). A pooled sensitivity and specificity of miR-92a were 76% (95% CI: 72%–79%) and 64% (95% CI: 59%–69%) in the diagnosis of CRC patients, respectively (Figure 2A). Its PLR and NLR in diagnosis CRC were 2.36 (95% CI: 1.56–3.58) and 0.32 (95% CI: 0.20–0.51) separately (Figure 2B). The summary DOR (Figure 2C) and the area under SROC were 8.05 (95% CI: 3.50–18.56) and 77.20% (Figure 3), suggesting a moderate diagnostic accuracy of miR-92a for CRC diagnosis.

### Meta-regression

Because the heterogeneity generated by non-threshold within the studies can be obviously observed in the forest plot of diagnosis index (as shown in Figure 2), we attempted to explain this heterogeneity by exploring the study characteristics, such as age, TNM stage, specimen numbers, using meta-regression. Unfortunately, no satisfactory clues were found.

### Publication Bias

**Figure 4:**
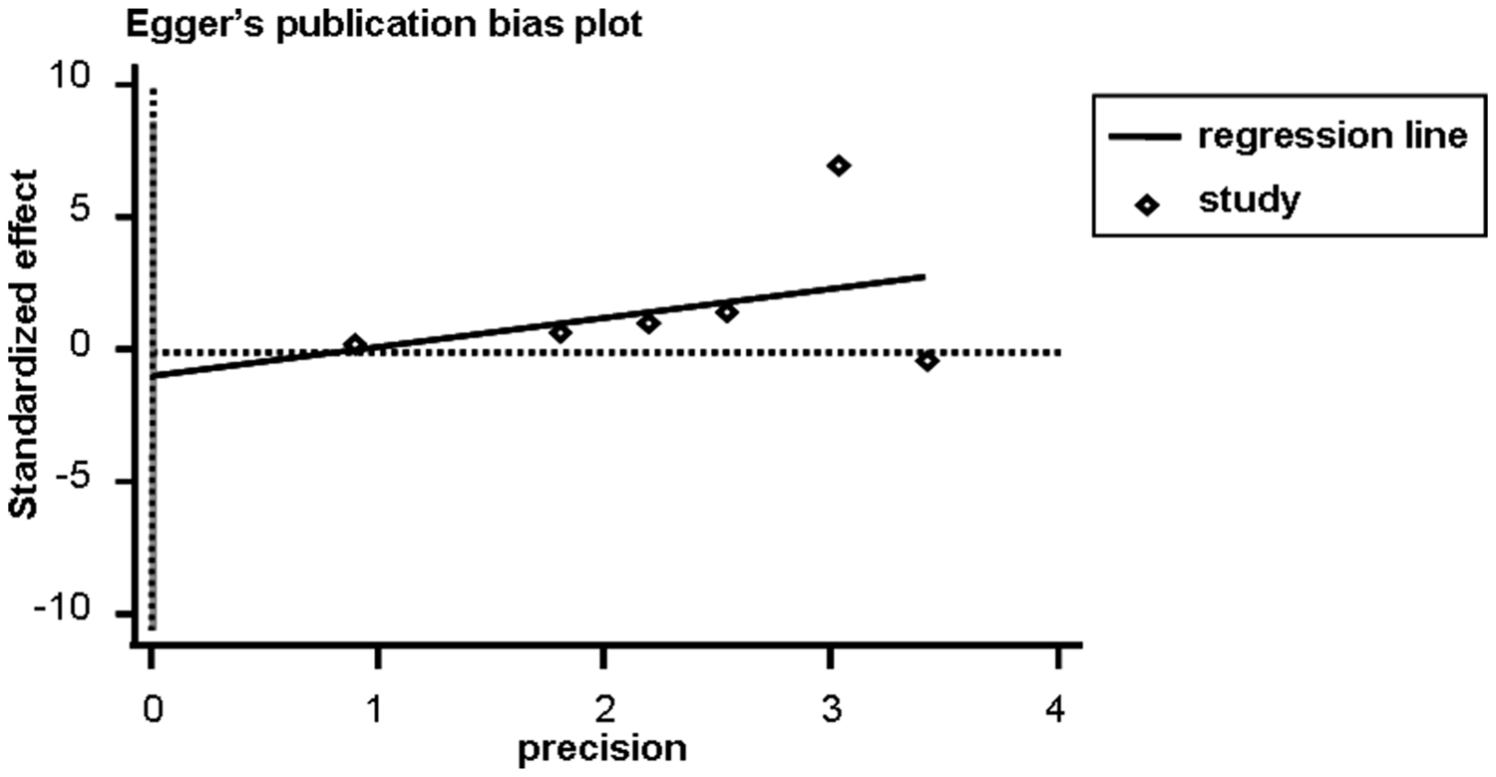
Publication bias from Egger’s test is shown by funnel plots. It is performed by funnel plot. Every point represents one study and the line is the regression line. It shows no publication bias exists.

The publication bias is recognized as another influent factor to the diagnosis accuracy [28]. The Begg’s test and Egger’s test were used in this meta-analysis. The *P* value is 1.000 for Begg’s test and 0.812 for Egger’s test, which is more than 0.05 and suggests no publication bias exist among these included studies (Figure 4).

## Discussion

To our knowledge, there is no evidence-based evaluation for miR-92a as a novel biomarker to diagnosis CRC since it was first reported on the quantitative assessment in patients with CRC. In this meta-analysis, we found discrepant expression levels of miR-92a in plasma have certainly statistical significance between the CRC patients and the control individuals. As a conclusion, the miR-92a discriminated CRC from controls and yielded an AUC of 0.772 with summary 76% (95%CI: 72%–79%) sensitivity and 64% (95%CI: 59%–69%) specificity, suggesting its potential diagnosis value of CRC as noninvasive detection. The diagnostic odds ratio (DOR), as an index representing compactness between diagnostic efficiency and the cases, has excellent test performance with a extremely higher value [29]. AUC is regarded as the overall test performance, and optimal value is infinitely close to 1 [30]. In our study, the DOR value of 8.05 (95%CI: 3.50–18.56) and AUC of 0.772 prompt a moderate diagnostic accuracy for diagnosing CRC.

It is indispensable for any meta-analysis that potential sources of heterogeneity are examined, before one considers pooling the results of primary studies into summary estimates with enhanced precision [31]. Fortunately, there is no heterogeneity caused by threshold effect in our meta-analysis. However, even though heterogeneity caused by non-threshold effect exists, we can’t find the sources of heterogeneity by Meta regression because of the limitation of the article numbers. In a meta-analysis, an important concern is the selection of studies. There is a risk that publication bias might adversely affect the reliability of the conclusions of the meta-analysis if the sampling is restricted to published studies, because published studies tend to the positive conclusion. [32]. However, publication bias is absent in our analysis with a smaller number of primary studies as well.

As a potential biomarker in the diagnosis of CRC, miR-92a has several obvious advantages.

Firstly, miR-92a is a stable biomarker. Retrospectively, a novel class of small regulatory RNAs, first described in 1993 by Lee et al. [33], has been the focus of intensive investigations. Mounting evidence has demonstrated the crucial functions of miRNAs in cancer initiation, progression and metastasis [7], [8]. Remarkably, recent literatures have confirmed that miRNAs can enter into the circulation system including blood and other body fluids [34]–[36], which have been speculated to be released from broken cells [37]. The discovery of miRNAs and its existence in blood have broken new ground for screening of cancer. Furthermore, endogenous plasma miRNAs exist in a form that is resistant to plasma RNase activity, which mean miRNAs in plasma remain largely intact and are indeed quite stable for detection [36].

Secondly, miR-92a is a non-invasive and convenient biomarker. Colorectal cancer is, if detected early, a highly curable disease. It were certified that the decrease in CRC incidence rates is largely duo to colonoscopy screening and the removal of precancerous lesion [38], however, the wide clinical application of this procedure is mostly limited by the invasive, unpleasant, and inconvenient nature [5]. Oppositely, as potentially powerful cancer biomarkers, circulating miRNAs have striking advantage in convenience, compliance and noninvasive [35]. Referring to FOBT, the notable lower sensitivity (23.9%) [4]) compared to miR-92a (sensitivity 76%) makes it such difficult to diagnosis in early stage CRC. Besides, they also have no requirement for dietary restriction and meticulous collection. In conclusion, our meta-analysis inspire that differential expression of a single miRNA in plasma could discriminate CRC from normal, raising the possibility of using such markers to develop a non-invasive and rapid diagnostic test for CRC in the future.

Last but not least, miR-92a is a higher sensitivity biomarker. Over the past several years, many groups have devoted themslves to define signatures that predict CRC, but there is still no practical diagnostic biomarker for CRC with satisfactory sensitivity and specificity. Carcinoembryonic antigen (CEA), which has been the first blood marker proposed in connection with CRC [39], overall sensitivity varied between 43% and 69%, and the most other common tumor markers for CRC, overall sensitivity ranged 18% to 65% for Carbohydrate antigen 19-9 (CA19-9) and 30% to 55% carbohydrate antigen 242 (CA242) [40]. Thus miR-92a, as a promising, higher sensitivity and noninvasive biomarker, has a prominent advantage over other markers for screening of CRC.

Although our results are promising, there are several limitations in this meta-analysis. On the one hand, due to the clinical value of miR-92a has been explored in CRC only for recent year, small sample size is contained in our meta-analysis, and as a result, small-study effects are inescapable. So it is necessary to strengthen our conclusion by further validations of miR-92a in large cohort and in independent studies. On the other hand, the specificity 64% (95%CI: 59%–69%) is not satisfactory in our study. The miR-92a, as an important part of the mir-17–92 cluster locating at 13q, is among the best characterized miRNA oncogenes, whose genomic amplification or aberrant elevation are frequently observed in a variety of tumor types [8]. This characteristic might make it uncertain whether this marker is specific for CRC. Furthermore, there are still some biases in our mete-analysis. For design type of eligible studies, only two of these studies clearly claimed using a prospective design, and 4 other studies did not mention. The cut-off value to evaluate the miR-92a expression in the 6 studies was selected from ROC curve, which is generally accepted to optimize the overall test performance. Moreover, no studies unequivocally mentioned whether a blind design was used in the research.

In conclusion, our study demonstrates miR-92a has reasonable sensitivity and is a potential biomarker for CRC detection by statistics method. If validated in a large scale study, miR-92a might be useful as a noninvasive screening tool for clinical practice of CRC.

## Supporting Information

Checklist S1 **PRISMA 2009 Checklist.**
(DOC)
